# Interest of Fluorescence Derivatization and Fluorescence Probe Assisted Post-column Detection of Phospholipids: A Short Review

**DOI:** 10.3390/molecules15010352

**Published:** 2010-01-18

**Authors:** Hanadi Ibrahim, Eric Caudron, Athena Kasselouri, Pratrice Prognon

**Affiliations:** Groupe de Chimie Analytique de Paris-Sud, Faculty of Pharmacy, University Paris-Sud, EA 4041, IFR 141, 92296 Châtenay-Malabry, France

**Keywords:** phospholipids, fluorescence, derivatizing reagents, probe assisted post-column detection

## Abstract

Phospholipids are essential constituents of all living cell membranes. There are many analytical methods available for the quantitative and qualitative determination of phospholipids, but since these molecules lack chromophores, common absorbance based methods are of limited use. Beside mass spectrometry, some less specific approaches that are routinely used are evaporative light scattering detection or fluorescence, which exhibit sufficient sensitivity. Here, we focus on fluorescence, which remains an interesting way to quantify phospholipids. Two ways of detecting phospholipids by fluorescence are possible coupled with separation techniques such as thin layer chromatography (TLC), high performance liquid chromatography (HPLC) and capillary electrophoresis (CE): firstly, pre-column derivatization procedures and secondly, probe assisted post-column detection with suitable fluorescence reagents. In both cases, the common purpose is to increase the detection sensitivity. It is shown that, whereas pre-column derivatization is characterized by selectivity due to the chemical functionality of the analyte involved in the derivatization process, in supramolecular post-column derivatization, the selectivity only proceeds from the capacity of the lipid to involve supramolecular assemblies with a fluorescence probe. The aim of this review is to summarize available experiments concerning fluorescence detection of phospholipids. The interest and limitation of such detection approaches are discussed.

## Abbreviations

Abbreviation of phospholipid classes will follow essentially those of IUPAC-IUB-recommended nomenclature for lipids [70].

Glycerophospholipid polar head group are be designed as:

GPA: Glycerophosphatidic acid
GPSer: Glycerophosphatidylserine
GPIns: Glycerophosphatidylinositol
GPEtn: Glycerophosphatidylethanolamine
GPCho: Glycerophosphatidylcholine
GPGro: Glycerophosphatidylglycerol

(phosphatidyl uniquely designates a 1,2-diacyl species)

*sn*-1, *sn*-2 and *sn*-3 designates carbon atoms of the glycerol backbone, where “*sn”* refers to *stereospecifically numbered *(IUPAC-IUB Commission on Biochemical Nomenclature, 1978)

Individual molecular species in this review are: n:jk/s:t-GXP (16:0e/20:4-GPEtn), where n is the number of carbons in the *sn-*1 radyl group, and j is the number of double bonds in the *sn*-1 hydrocarbon chain; k represents the type of *sn*-1 linkage, where a =1-*O*-acyl, e = 1-*O*-alkyl ether, p = 1-*O*-alk-1’-enyl (plasmalogen); s is the number of carbon atoms; and t is the number of double bonds at the *sn-*2 radyl substituent. The accepted nomenclature for phospholipids results in long complex names, and this nonstandard abbreviation system has been found to be useful by the authors to describe the most common phospholipid molecular species found in biological system.

A general term for molecular species, but not exactly defining the fatty-acyl substituent, follows the form m:x-GPX (38:4-GPCho), where m is the total number of carbon atoms in *sn-*1 and *sn-*2 radyl groups, and x is the total number of double bonds in both radyl groups.

## 1. Introduction

### Lipids: A complex world

The role of lipids in cellular biochemistry is only partly understood, but the knowledge in this field is now rapidly expanding together with the development of the analytical tools for lipid study (lipidomics). Lipids are essential cellular constituents and play a variety of roles in cellular functions. Firstly, the majority of lipids form the bilayer cell membrane whose integrity and physical characteristics are vital for life processes by providing an environment necessary for the function and interactions of proteins. Secondly, lipids are the major energy store which can be rapidly accessed when needed. Thirdly, membrane lipids are the source of numerous second messengers generated by the actions of a variety of intracellular enzymes [1,2,3,4].

No universal definition of the term “lipid” is available, because neither chemical structures nor chemical functions can be used to characterize this class. A general definition of lipids could be “non-water soluble and soluble in organic solvents”. Cellular lipids can be roughly classified into two major groups, *i.e.* non-polar lipids and polar lipids. Non-polar lipids include predominantly triacylglycerols, cholesterol and cholesterol esters, which are small, weakly polar molecules exhibiting a dominant hydrophobic region. Polar lipids essentially consist of phospholipids, sphingolipids and glycolipids.

**Figure 1:**
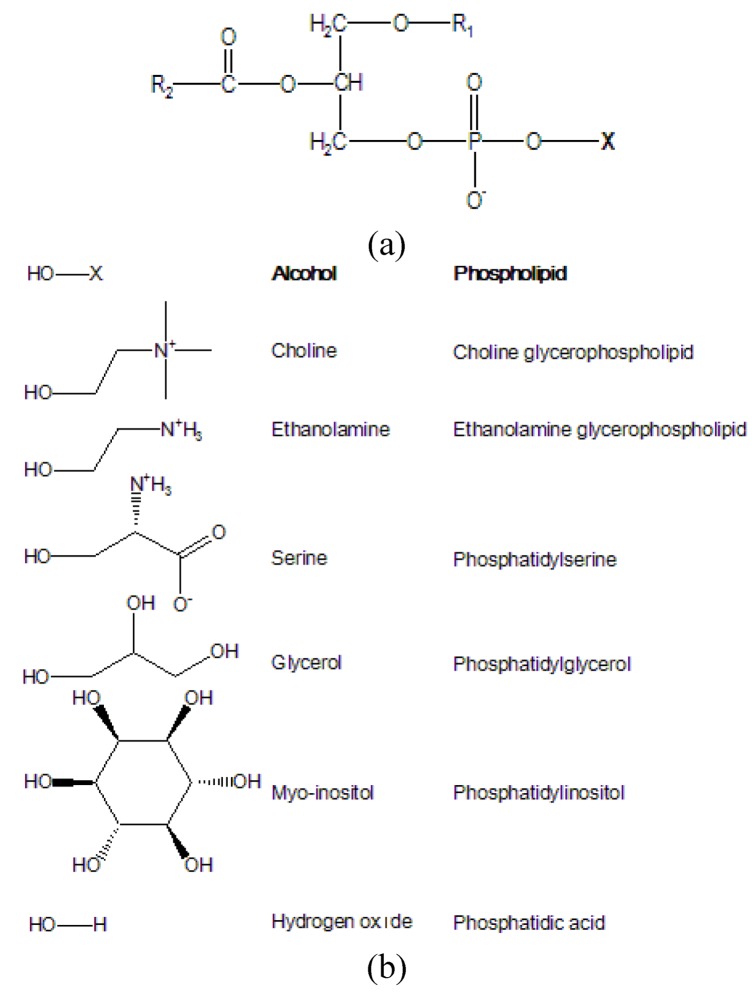
Basic phospholipid structure (a) and moieties of X (b).

This review will focus on the analysis of phospholipids, *i.e.,* lipids containing phosphoric residues. Phospholipids are amphiphilic molecules possessing polar head groups and non-polar lipid chains. There are two major classes of phospholipids: glycerophospholipids (or phosphoglycerids) that contain a glycerol backbone; the other is a sphingosine-based phospholipid, sphingomyelin. Glycerol-based phospholipids are predominant in eukaryotic cells and constitute 60 mol % of the lipid mass [5]. The general structure of glycerophospholipids is a glycerol backbone grafted in *sn*-3 with a phosphate ester group, a fatty acyl group at the *sn*-2 position, and a fatty acyl or alkyl group at the *sn*-1 position. Based on chemical differences (Figure 1-a) of the structures that are linked to the phosphate, phospholipids can be classified in a large number of classes (Figure 1-b).

The X group is typically choline, ethanolamine, serine, myo-inositol, glycerol and hydrogen oxide (water) (Figure 1-b). The corresponding classes are called choline glycerophospholipid, ethanolamine glycerophospholipid, *etc*.

**Figure 2:**
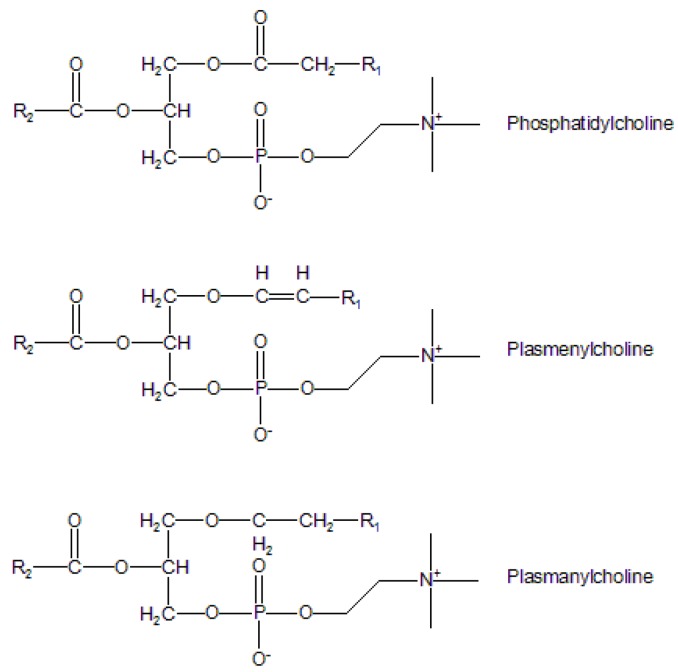
The subclasses of choline glycerophospholipids. Classification based on the linkage of aliphatic chain at the sn-1 position of the glycerol backbone.

Based on the covalent nature of the linkage of the aliphatic chain at the *sn*-1 position of the glycerol backbone, each phospholipid class is further subdivided into three subclasses: phosphatidyl, plasmenyl and plasmanyl, corresponding to ester, vinyl ether and alkyl ether linkages, respectively (Figure 2). Among membrane lipids, the phosphatidyl subclass of phospholipids is predominant. Nevertheless, the presence of these glycerophospholipid subgroups varies with tissues, e.g., ether lipids are common in inflammatory cell membranes [6], and plasmalogen species are common components of cardiac tissue [7].

The complexity of phospholipid molecules is due not only to the variation of polar groups (classes) and the different aliphatic chains bonded in *sn*-1 (sub-classes) but also to the length, number and location of double bonds of the two aliphatic chains of the phospholipid skeleton (molecular species). As an example, the length of grafted fatty acids can vary from 14 to 22 carbon atoms and can contain between zero and six double bonds [8]. Further diversity also arises from asymmetry of the cell-membrane bilayer: The inner and outer leaflets of the membrane have different phospholipid contents, with the inner leaflet highly enriched in glycerophosphoethanolamine and glycerophosphoserine. This bilayer asymmetry has also been reported as an important feature of cellular activation [9]. Some phospholipids present in very low levels are called minor phospholipids, such as platelet activating factor (PAF) or lysophosphatidic acid, but these lipids nevertheless play pivotal roles in immune regulation and defense mechanisms and in the maintenance of homeostasis in living systems.

## 2. Detection of Phospholipids: An Analytical Challenge

Chromatographic methods have been commonly used for the isolation, separation and quantification of phospholipids in the presence of other lipids in tissue samples. Thin layer chromatography (TLC) has classically been the simplest technique applied to lipid analysis, but the method suffers from poor reproducibility, tedious automation and quantitation of low precision due to technical issues of homogeneity of plate scanning [10,11,12,13]. High performance liquid chromatography (HPLC) is the most frequently used analytical method for phospholipid analysis and is superior to TLC since it provides higher resolution and reproducibility [10,12,13]. In addition, sample preparation and clean-up procedures are also simpler and less time-consuming [13,14]. Capillary electrophoresis (CE) provides an alternative to HPLC for the analysis of phospholipids as a result of its high separation efficiency, versatility of application and easy to use instrumentation. While CE is a well established technique for analyzing a variety of substances such as amino acids, peptides, proteins and DNA [12], its use for phospholipid analysis is rather limited, probably because of low water solubility and low UV absorbance, leading to high limits of detection (LOD).

Phospholipids lack structural groups or moieties that could be used for selective detection, *i.e.* lack of chromophores, and this has been a major obstacle for identification and quantification. In any case, this drawback can be easily overcome by the use of mass spectrometry which is not limited by this aspect. Mass spectroscopy, the most sensitive and specific detection technique [5,8,13,15] remains relatively expensive and less widely used in spite of its versatility. Unfortunately, the investment and maintenance of such equipment have prompted analysts to continue exploring others possibilities. Among them, quantification of phospholipids is feasible with detectors providing a reproducible mass or molar response such as evaporative light scattering detection (ELSD) [16]. It is also of major interest to obtain a maximally sensitive response that is linear over the broadest possible range as with fluorescence. Nevertheless, no commercially available detector fully meets all these requirements. Consequently, the compromise involves selecting the most appropriate detection system for a specific problem.

In this context, various detectors can be envisaged for use in conjunction with HPLC and CE for the analysis of phospholipids. The first is a refractive index detector whereas well suited for qualitative and quantitative work [17], is limited by average sensitivity and its high degree of response variability due to its dependence on temperature, pressure and solvent flow-rate [17,18,19]. Refractive index measurements and short-wave UV detection [20,21] systems were employed in early HPLC of phospholipids [18]. This type of detection requires extensive calibration and is subject to serious solvent restrictions, especially in the case of UV detection with low sensitivity [18].

It has recently been shown that phospholipids can be suitably detected by universal detection systems such as a light scattering detector [16], charged aerosol detector or mass spectrometer. Development of the light scattering detector, and more recently the charged aerosol detector (so called Corona-CAD detector) is an apparent breakthrough for the detection of non-UV absorbing compounds such as phospholipids [19,22,23,24]. This is true even though there are disadvantages with this type of detector such as its being a destructive technique and yielding a non-linear response, making linear calibration curves inaccurate [25].

This is why an alternative method for the quantitation of non-UV absorbing lipids such as phospholipids involves derivatization using a suitable chromophore or fluorophore in order to improve the sensitivity and selectivity of detection, regardless of the separation technique used, *i.e.,* TLC, HPLC or CE. An ideal derivatizing reagent reacts rapidly and quantitatively with the analytes while the resulting derivative exhibits an enhanced signal in comparison to the non-derivatized species [26]. For obvious reasons of specificity and sensitivity, fluorescence is preferred, especially in the analysis of complex samples.

Fluorescence detection methods applied to phospholipids can be broken down into two main categories: covalent pre-column derivatization and fluorescence probe assisted post-column detection methods.

### 2.1. Pre-column derivatization of phospholipids

Most fluorescence reagents have been developed for derivatization carried out prior to chromatographic separation. In this case, care must be taken to ensure the stability of the reaction product during its storage before HPLC or CE injection. Fluorescence chemical derivatization provides both selective and sensitive detection of analytes, and appears to be very useful in phospholipid analysis [27,28].

### 2.2. Fluorescence probe assisted post-column detection of phospholipids

In post-column detection, the reaction process is continuously performed after chromatographic separation. It can often be conducted when the covalently derivatized product is unstable, and the derivation rate is compatible with the chromatographic run.

In the present case, the reported post-column detections involve a supramolecular assembly between a fluorescence probe and the eluted phospholipids. This non-covalent reaction must be compatible with the duration of the separation process and is based on the change in fluorescence characteristics of the fluorescence probes with phospholipids. We will now focus our attention on pre-column derivatization or post-column fluorescence probe assisted detection of phospholipids separated by TLC, HPLC or CE. 

## 3. Pre-Column Fluorescent Covalent Derivatization of Phospholipids

In this section, the chemical fluorescence derivatization of grafted amino, hydroxyl or carboxyl phospholipids is described and operating conditions and chemical structures of the reagents cited in Section 3.1 are summarized (Table 1, see also Figure ).

Most phospholipids were analyzed by HPLC and CE is relatively less widely used. A reactive chemical functionality is required to covalently derivatize phospholipids. The derivatization site for aminophospholipids such as phosphatidylserine or ethanolamine glycerophospholipids is usually the amino group. For phospholipids with no free amino group or other reactive groups, a hydroxyl function is generated after enzymatic or chemical hydrolysis, especially for glycerophospholipids.

### 3.1. Derivatization without phospholipid hydrolysis

#### 3.1.1. Amino phospholipids

1-Dimethylaminonaphthalene-5-sulfonyl chloride (DNS-chloride) [29] or succinimidyl 2-naphthoxyacetate [32] are well known reagents of primary and secondary amines used to form fluorescent derivatives and are well suited for amino phosphoglycerides. In the publications cited, DNS derivatives of rat brain phophatidylethanolamine, lysophophatidylethanolamine, phophatidyl-serine and lysophophatidylserine were analyzed by HPLC at concentrations as low as 20 pmol injected (limits of detection). DNS derivatives were prepared by reacting phophatidylethanolamine with DNS-Cl [30,31] according to Chen *et al.* [29].

CE is a powerful tool for the analysis of biomolecules but there are a very few publications dealing with phospholipid analysis and fluorescence detection. A micellar electrokinetic capillary chromatography (MECC) method with laser-induced fluorescence (LIF) was developed for the analysis of amino-containing phospholipids, including phosphatidylethanolamine, phophatidyserine, lysophosphatidylethanolamine and lysophophatidyserine [12]. 3-(2-furoyl)quinoline-2-carboxaldehyde (FQ) was successfully used to label these phospholipids but 4-fluoro-7-nitrobenzofurazan (NBD-F) produced a fluorescent product from only lysophosphatidylethanolamine and phosphatidyl-ethanolamine and surprisingly no signal was observed with lysophosphatidyserine and phosphatidyserine. A laser operating at 488 nm provided fluorescence excitation and emission fluorescence was collected at 630 nm and 535 nm for FQ and NBD-F derivatives, respectively. This method, using an efficient separation with a methyl-β-cyclodextrin-modified MECC system, provided limits of detection (Table 1) from 0.18 to 1.1 fg (10^-9^–10^-10 ^M), which was four to five orders of magnitude higher than CE—Indirect photometric detection applied to the determination of phospholipids [37].

Phenylisothiocyanate (PITC) has often been used as an amino acid and protein derivatization reagent to form stable phenylthiocarbamyl derivates with primary and secondary amino groups. It has been shown that PITC can also be used to detect phosphatidylethanolamine and phosphatidylserine; phospholipids containing amino groups [38].

A fluorescent reagent, *ortho*-phtalaldehyde (OPA) was used because of its specific reaction with primary amino groups to derivatize sphingosine-1-phosphate and other sphingoid based 1-phosphates [33,34]. The OPA reagent, containing OPA and mercaptoethanol in methanol at pH 10.4, has been used to form fluorescent isoindole derivatives from sphingosine followed by HPLC separation. The quantitative determination of sphingosine-1-phosphate in plasma used solid phase extraction followed by an automated reversed-phase gradient HPLC column-switching system for pre-column derivatization with OPA [34]. More recently, He *et al*. described an HPLC method to quantify sphingosine-1-phosphate and other sphingoid base 1-phosphates in biological samples after derivatization with naphthalene-2,3-dicarboxaldehyde (NDA) [35]. The limit of detection was 20.9 fmol injected and the fluorescent derivatives were monitored with excitation and emission wavelengths of 252 nm and 483 nm respectively. This procedure used a stable and highly sensitive fluorogenic reagent. NDA reacts specifically with primary amino groups in the presence of CN^-^ to form 1-cyanobenz[f]isoindole derivatives. Moreover, this method requires only a single lipid extraction and does not require dephosphorylation of sphingosine.

**Table 1:** Fluorescence derivatizations used in HPLC and CE with DNS-chloride: 1-dimethyl-aminonaphthalene-5-sulfonyl chloride, FQ: 3-(2-furoyl)quinoline-2-carboxaldehyde, NBD-F: 4-fluoro-7-nitrobenzofurazan, OPA: ortho-phtalaldehyde and NDA: napthalene-2,3-dicarboxaldehyde. Cl : naphthalene.

Analyte	Matrices	Derivatizing reagent	Separation Type	Temperature condition (°C)	Reaction time	λ_ex_-λ_em_ (nm)	Limit of detection	Refs.
*Phospholipids with amino group*								
GPEtn, lyso-GPEtn, GPSer, lyso-GPSer	Rat brain	DNS-chloride	HPLC	50	3h	342-500	20 pmol	[29]
GPEtn	Egg	DNS-chloride	HPLC	50	2-3h	360-> 420	-	[30,31]
GPEtn, lyso- GPEtn, GPSer, lyso- GPSer	Rat brain	succinimidyl 2-naphtoxyacetate	HPLC	Room temperature	2h	228-342	2 pmol	[32]
GPEtn, lyso- GPEtn, PS, lysoPS	-	FQ	MECC	55	15min	488-630	0.65-1.1 fg	[12]
PE, lysoPE, PS, lysoPS	-	NBD-F	MECC	55°C	5min	488-535	0.18-0.87 fg	[12]
Sphingoid base 1-phosphates	Human plasma, serum, platelets	OPA	HPLC	Room temperature	20 min	340-455	< 5 pmol	[33]
Sphingosine 1-phosphate	Human plasma	OPA	HPLC	Room temperature	20 min	340-455	< 50 ng/mL	[34]
Sphingosine 1-phosphate	Plasma (human, horse, mouse), mousse tissues	NDA	HPLC	50°C	10 min	252-483	20.9 fmol	[35]
*Phosphatidic acid*								
GPA	Egg	DNS-ethanolamine from DNS-Cl	HPLC	Room temperature	2-3h	360-420	-	[31]
GPA	Rabbit platelets	3-(9 anthroyl) diazo-2-propene	HPLC	4°C	8h	254-430	0.05ng/mL	[36]

**Figure 3:**
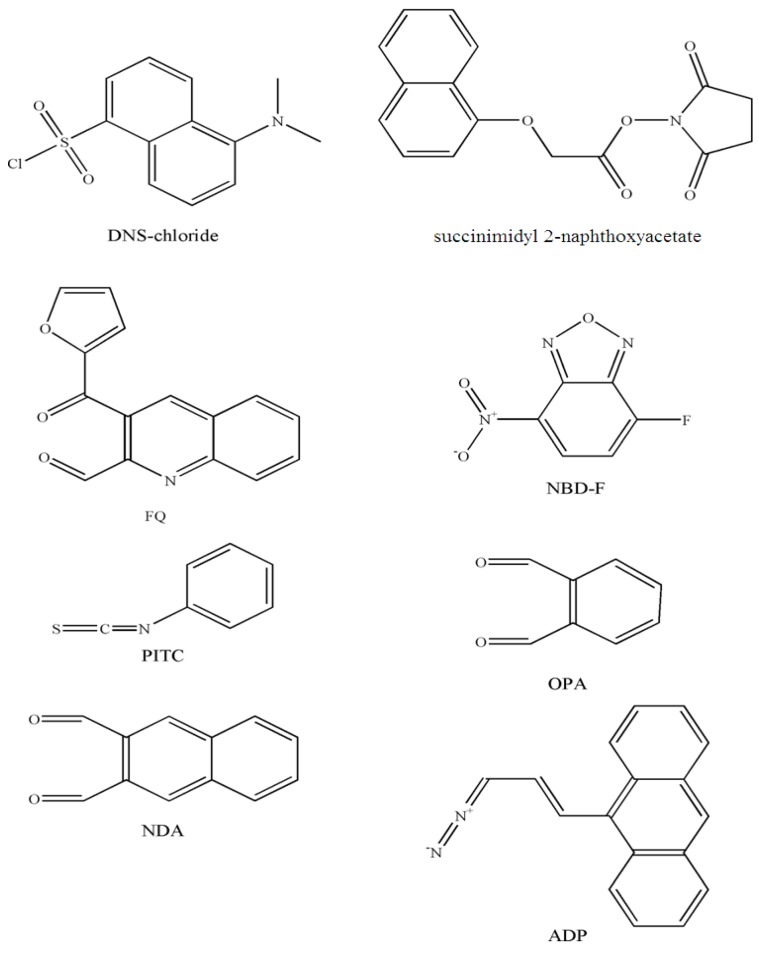
Chemical structures of the reagents cited in Section 3.1.

#### 3.1.2. Phosphatidic acid

Phosphatidic acids are the common structural moiety of all glycerophospholipids. It is the precursor of diacylglycerol, which is required in the *de novo* synthesis of phosphatidylcholine or phosphatidylethanolamine. As an important metabolic intermediate, negatively charged weakly concentrated phosphatidic acid coexists with large amount of glycerophospholipids in cell membranes. As a result, the analysis of phosphatidic acids is difficult and requires a high degree of sensitivity. The free acid moiety can be tagged with fluorophores to form fluorescent derivatives. The reaction is thus an esterification of phosphatidic acid with a mixture of 5-dimethylaminonaphthalene-1-sulfonyl chloride, a derivative of ethanolamine (DNS-ethanolamine), 2,4,6-triisopropylbenzene sulfonyl chloride, triethylamine and pyridine to form a monoanionic fluorescent derivative: DNS-phosphatidylethanolamine DNS-PE [31]. The derivatization of phosphatidic acid with 3-(9-anthryl) diazo-2-propene (ADP) yields a highly fluorescent compound: di[3-(9-anthryl) diazo-2-propenyl]-phosphatidic acid [36].

### 3.2. Derivatization after phospholipid hydrolysis

**Figure 4:**
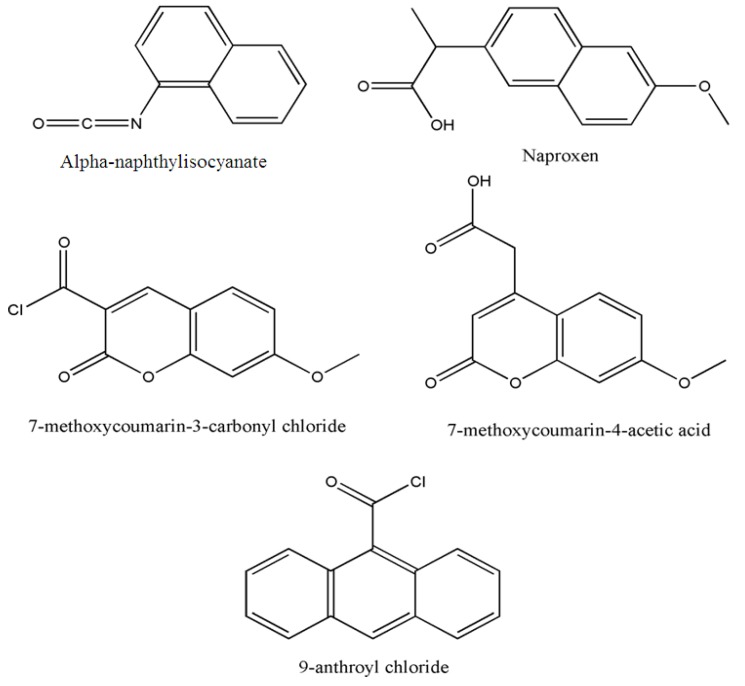
Chemical structures of the reagents described in Section 3.2.

In this section, the chemical fluorescence derivatization after phospholipid hydrolysis is described. Operating conditions and chemical structure of the reagents described are summarized (Table 2 and Figure 4).

For phospholipids free of amino or hydroxyl groups, their hydrolysis before derivatization is necessary in order to release a reactive functionality. A large number of derivatization procedures have been used after hydrolysis of phospholipids, especially for glycerophopholipids. One example is enzymatic or chemical hydrolysis of glycerophopholipids to form diacylglycerols, which are acylated with fluorescence chromophores on the free OH group.

#### 3.2.1. Diacylglycerols obtained after hydrolysis by phospholipase C

Phosphatidylcholines obtained from rat liver have been separated by two-dimentional TLC. Spots of phosphatidylcholine extracted from plates form alpha-naphthylisocyanate derivatives of diacylglycerols obtained after hydrolysis by phospholipase C and reaction with a 200 fold-molar excess of alpha-naphthylisocyanate and a 4-fold excess of 1,4-diazabicyclo(2,2,2)octane [39]. DNS-phosphatidylethanolamine was synthesized from diacylglycerols by the esterification of the hydroxyl group with the phosphoric acid of DNS-ethanolamine phosphate (prepared by reacting DNS-Cl and ethanolamine phosphate) and the phosphoric acid of phosphatidate from the enzymatic hydrolysis of egg phosphatidylcholine or phosphatidylethanolamine [30]. Another sensitive analysis method for diacylglycerophospholipids was developed. In this work, molecular species of diacylglycerophospholipids obtained after enzymatic hydrolysis from rat brain cerebrum and cerebellum and diacylglycerol were derivatized by naproxen chloride, obtained from naproxen (6-methoxy-alpha-methyl-2-naphthaleneacetic acid) in the presence of 4-dimethylaminopyridine were separated by HPLC and quantified by absorbance at 230 nm or by fluorescence (excitation at 332 nm and emission at 352 nm). The high molar absorption coefficient of naxopren derivatives (53,000 L·mol^-1^·cm^-1^ at 230 nm) led to absorbance detection of less than 10 pmol. A 10-fold increase in sensitivity was obtained with a fluorescence detection system, resulting from the fluorescent properties of adducts [40]. Anthroyl diacylglycerol derivatives obtained after phospholipase C treatment have also been used for phospholipid molecular species of phosphatidic acid and phosphatidylcholine from rat thymocytes [41] or phospholipids from Chinese hamster V79-R [45]. The sensitivity of detection enabled measurements at the subpicomole level, 10 to 100-fold higher than that available with UV detection of similar chromophores [41]. The authors also reported the feasibility of application to other biomolecules containing an available hydroxyl group, *i.e.*, hydroxyleicosatetraenoates (HETEs).

**Table 2:** Fluorescence derivatizations after hydrolysis used in HPLC and CE.

Analyte	Derivatizing reagent	Matrices	Separation	Temperature condition (°C)	Reaction time	λ_ex_- λ_em_ (nm)	Limit of detection	Refs.
*Hydrolysis by phospholipase C*								
GPCho	alpha-naphthylisocyanate	Rat liver microsomes	HPLC	85	2 h	280-360	10 pmol	[39]
GPCho, GPEtn	DNS-ethanolamine phosphate from DNS-Cl	Egg	HPLC	60-80	24 h	360-420	-	[30]
GPEtn	Naproxen chloride	rat brain cerebrum and cerebellum	HPLC	80	15 min	332-352	1 pmol	[40]
GPCho, GPA	9-anthroyl chloride	Rat thymocytes	HPLC	60	10 min	360-460	0.1 pmol	[41]
PAF	7-methoxycoumarin-3-carbonyl chloride or 7-methoxycoumarin-4- acetic acid	Human leucocytes	HPLC	-	-	-	100 pg	[42]
*Hydrolysis by phospholipase D*								
GPCho	DNS-ethanolamine from DNS-Cl	Egg	HPLC	Room temperature	2-3 h	360-420	-	[31]
*Hydrolysis by alkaline phosphatase*								
Sphingoid base 1-phosphates	OPA	Serum, cultured cells, rat tissues	HPLC	Room temperature	20 min	340-455	0.5 pmol	[43]
Sphingosine 1-phosphate and dihydro Sphingosine 1-phosphate	OPA	Cultured cells, plasma	HPLC	Room temperature	20 min	340-455	<0.5 pmol	[44]

Platelet-activating factors (PAF), 1-*O*-hexadecyl- and 1-*O*-octadecyl-2-acetyl-sn-glycero-3-phosphatidylcholine were hydrolyzed with phospholipase C and the resulting hydrolyzed products were derivatized with 7-methoxycoumarin-3-carbonyl chloride or 7-methoxycoumarin-4-acetic acid to form 7-methoxycoumarin ester derivatives for detection. The limit of detection of derivatives was about 100 pg [42].

#### 3.2.2. Phosphatidic acid obtained after hydrolysis by phospholipase D

Phosphatidic acids were obtained by the enzymatic hydrolysis of egg phosphatidylcholine by phospholipase D. DNS-phosphatidylethanolamine was then prepared by esterification between the hydroxyl group of DNS-ethanolamine (prepared by reacting DNS-Cl and ethanolamine) and the phosphoric acid moiety of phosphatidate [31].

#### 3.2.3. Sphingoid base obtained after hydrolysis by alkaline phosphatase

This publication described an analytical method for the simultaneous measurement of sphingoid base 1-phosphates and sphingoid bases from a variety of biological samples. The two-step method of sample pretreatment involved the enzymatic dephosphorylation of sphingoid base 1-phosphates by alkaline phosphatase (APase), followed by the HPLC analysis of *o*-phthalaldehyde (OPA), made possible by its specific reaction with derivatives of primary amino groups of the sphingoid bases released [43]. More recently, a selective enrichment of sphingosine-1-phosphate and dihydro sphingosine-1-phosphate from biological samples was achieved by Fe^3+^ gel immobilized metal affinity chromatography. Sphingosine-1-phosphate eluted was then dephosphorylated, derivatized with *o*-phatalaldehyde (OPA) and detected by fluorescence after HPLC separation [44]. This procedure, however, requires special columns and time-consuming lipid extraction, as well as a dephosphorylation step. The well-known instability of OPA is also an important issue.

As discussed above, the hydrolysis strategy is very useful for phospholipid and its subclass analysis. For those phospholipids that have no free amino or hydroxyl groups, it is better to first hydrolyze them and then derivatize with various reagents. This method appears to be the most commonly used.

## 4. Probe Assisted Fluorescence Detection after Native Separation of Phospholipids

In Sections 4.1. and Sections 4.2, the chemical structure of the reagents described are summarized (Figure ).

### 4.1. Fluorescence detection after TLC

Since the 1960s, one- or two-dimensional TLC or high-performance-TLC (HPTLC) has often been used to separate phospholipid extracts. Phospholipids are visualized and quantified on the TLC plate by using spray reagents after separation. Detection was colorimetric (or densitometric). TLC has been routinely used for the isolation and identification of phospholipids from biological samples.

A TLC detection reagent, such as 1-anilinonaphthalene-8-sulphonate (1,8-ANS) was used for the quantitative determination of nanogram quantities of phosphatidylcholine and sphingomyelin [46]. 0.2% 2’,7’-dichlorofluorescein 0.2% sprayed to visualize phospholipid bands, which appeared as fluorescent spots under UV light at 366 nm [47,48].

Primuline is common in lipid dye research. It binds non-covalently to lipid structures and thus enables direct evaluation in TLC. Phospholipids were visualized by spaying with a solution of primuline dye (direct yellow 59). Individual phospholipids were detected as violet spots under UV light at 366 nm and by fluorescence spectroscopy [49,50]. Richter *et al.* [50] showed that primuline UV absorption was affected by treatment with phosphatidyl ethanolamine in the presence of HOCl due to fading of the dye, but this did not affect fluorescence properties [50].

2,5-bis-2-(5-*tert*-Butyl)benzoxazolylthiophene (BBOT) has been used for the analysis of phospholipids such as phosphatidylcholine, lysophosphatidylcholine or sphingomyelin. Bright fluorescing spots of lipids can be seen against a dark and only weakly fluorescing background in UV light at 366 nm when the plate was held at 50 °C for 1 h after probe application [46]. The limit of detection is 800 ng of phosphatidylglycerol [51], and 20 ng or less of substance following circular HPTLC [46], without fluorescence bleaching over 24 h. The two fluorescent probes used for TLC visualization are suitable for the fluorimetric detection of phospholipids because of its low fluorescence background on TLC plates and its significantly enhanced quantum yield of fluorescence in the presence of phospholipids. HPLC is now clearly preferred over TLC because of higher resolution and reproducibility. 

### 4.2. Post-column detection after HPLC

An alternative phospholipid detection technique is based on post-column fluorescence derivatization using a supramolecular combination of a fluorescence probe and eluted lipid. Fluorescence probes are small molecules which undergo changes in their fluorescence characteristics after interaction with their environment, *i.e.,* solvent, macromolecules, fluidity, pH, *etc*., as a result of non-covalent interactions [52].

The detection of supramolecular assemblies was proposed for the analysis of phospholipids after HPLC separation with BBOT, a probe used for TLC detection [51]. This probe appears to be specific for phospholipids [46]. In the absence of phospholipids, low fluorescent response was observed when chloroform, THF or methanol were mixed with BBOT, but a drastic fluorescence enhancement was observed with phospholipids with bathochromic shift: 372 and 440 nm for excitation and emission fluorescence respectively. The low background fluorescence of BBOT in organic solvents was of interest because of a low post-column dilution. Indeed, the optimum mobile phase/post-column flow rate ratio was set at 4. Despite this low post-column dilution, the limit of detection with BBOT is about 10 µg/mL, representing 300 ng of solute injected.

**Figure 5:**
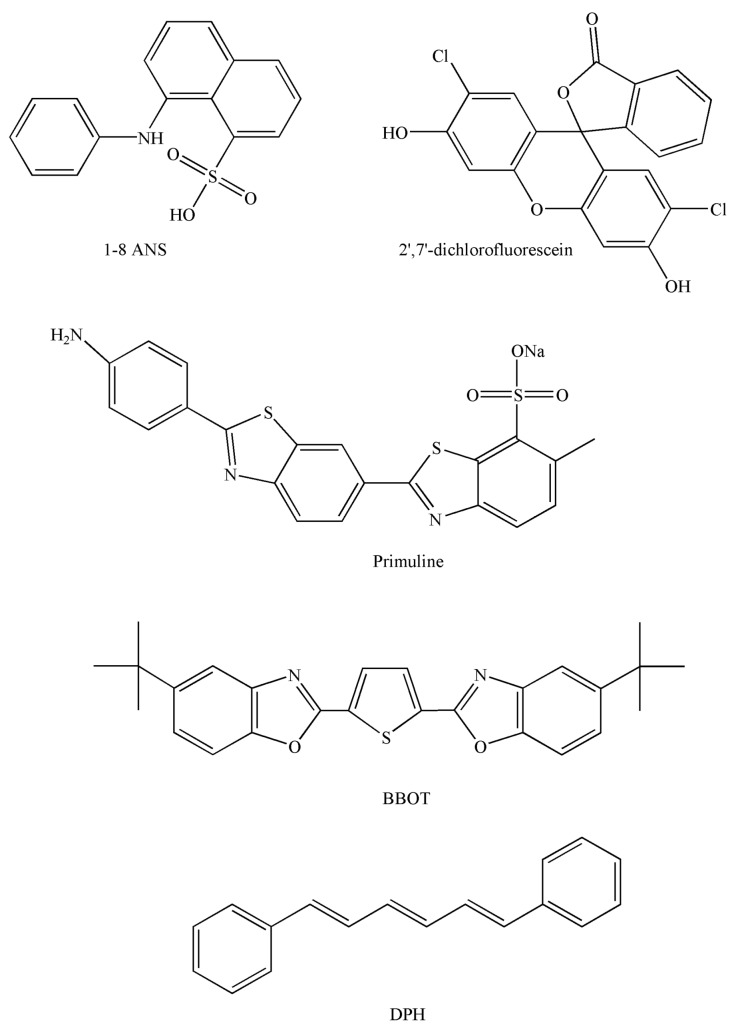
General chemical structures of molecular probes used for TLC and HPLC post-column detection.

This is not comparable with 1,6-diphenyl-1,3,5-hexatriene (DPH) in terms of the limit of detection, which was about 1 µg/mL [53,54]. DPH is in fact the most widely proposed molecular probe for the post-column fluorescence derivatization of lipids after liquid chromatographic separation because it exhibits a very weak fluorescence in water and a high quantum yield of fluorescence in a hydrophobic environment. DPH is a polyene hydrocarbon compound characterized by a stable all-*trans* configuration that is known to be the exclusive configuration that is responsible for the native fluorescence of DPH in solution [55]. DPH can be dispersed in aqueous media to form microaggregates which are practically devoid of fluorescence [56,57]. When such an aqueous dispersion is mixed with lipid-containing systems, the probe is molecularly incorporated in the hydrophobic domain and exhibits a drastic increase of fluorescence emission. DPH is localized almost exclusively in the hydrocarbon core of the lipid [55,58,59] *i.e.,* the lipid chain domain for PL. An application of the use of DPH has been described for lipid detection due to its low fluorescence background in water and its strong fluorescence enhancement in lipid environments such as phospholipid vesicles [59] or parenteral nutrition admixtures [60]. This property of DPH is, also, used for lipid detection after liquid chromatography separation due to supramolecular assembly between the probe and the eluted lipids and it could also have applications for phospholipid analysis [53,54,61,62,63,64].

In order to obtain better spectroscopic insight into the nature of supramolecular assemblies during post-column detection, dimyristoylphosphatidylcholine (14:0/14:0-GPCho) as a model of phosphatidylcholine was studied by steady-state fluorescence measurements [65]. These results are in agreement with a dimyristoylphosphatidylcholine lipid bilayer organization. The outer diameter of dimyristoylphosphatidylcholine nanoparticles was 20 nm, compatible with a small unilamellar vesicle (SUV) with about 2600 dimyristoylphosphatidylcholine and 26 DPH, *i.e.,* 1 DPH/100 dimyristoyl-phosphatidylcholine. This liposome organization is highly probable for other phospholipids as well.

The post-column fluorescence detection system consisted of an HPLC pump connected via a pulse damper to a T-connector on line with reactor tubing just before the fluorescence detector. The post column addition reagent was an aqueous solution containing a non-ionic surfactant and the molecular probe. Five major factors influence post-column detection: surfactant (structure and concentration), probe, post-column flow-rate (or post-column/chromatographic flow-rate ratio), post column tubing length (or reaction time) and post column system temperature.

### 4.3. Surfactant

The surfactant was used with DPH probe and not with BBOT. This can be explained by a lower water solubility for BBOT than DPH. A surfactant is in fact mandatory to limit the build-up of background fluorescence due to DPH adsorption on tubing and the flow cell [62] and facilitates lipid and probe solubilization, as well as the molecular probe-lipids assembly [54]. Different non-ionic surfactants have classically been added to aqueous solutions of DPH: Tween 20 [61,62], Triton X-100 [59] and Brij 35 [54,63,64,66]. For practical reasons, surfactants were used below their critical micellar concentration (CMC) in order to avoid direct DPH fluorescence enhancement. Optimizing the Brij 35 concentration in the post-column mixture in fact showed a significant increase of fluorescence when the concentration was close to the CMC [66]. The CMC value of Brij 35 using DPH as a fluorescence probe was found to be 77 µM in water (0.031% v/v) and in a hydro-organic mixture [65]. Above the CMC, background noise increases and the signal-to-noise ratio decreases. This justifies using a surfactant concentration lower than the CMC in order to limit DPH adsorption on tubing with low background fluorescence.

### 4.4. Probes

An ideal molecular probe must have a very low fluorescence background in the post column mixture in the absence of lipids in order to minimize background noise and provide a high fluorescence signal in a lipid environment, *i.e*., high quantum yield of fluorescence in an apolar environment. Lipid-probe combination kinetics must be compatible with the limiting length of reacting tubing and to minimize extra-column band broadening. BBOT appears to be an interesting probe due to its low background fluorescence in an organic environment, which limits post-column dilution. Nevertheless, it shows a high limit of quantification, probably due to its low quantum yield in lipid media. Limits of quantification were equal or lower with DPH as molecular probe (Table 3). The major difference with BBOT is that DPH is fluorescent in organic media. In most publications, LC separation of phospholipids is carried out in organic media [18], although efficient detection with DPH is better in a highly aqueous environment. Since water is necessary to limit DPH background fluorescence and to induce lipid self-association required for DPH insertion, the post-column flow must be high and lead to a final predominant organic environment [65]. Unfortunately, the higher the post-column flow, the greater the post-column dilution.

**Table 3:** Fluorescence post-column derivatizations used in HPLC.

Refs.	[51]	[53]	[61]	[62]	[­63]	[64]	[54]
Molecular probe	BBOT	DPH	DPH	DPH	DPH	DPH	DPH
Concentration before	0.023	0.15	0.45	0.00045	0.45	10.5	3.35
(and after) mixing (µmol/L)	-0.006	-0.13	-0.34	-0.00025	-0.37	-7	-2.79
λex-λem (nm)	372–440	365–460	340–460	340–460	340–460	340–460	350–430
Surfactant	-	-	Tween 20	Tween 20	Brij 35	Brij 35	Brij 35
Concentration (v/v)			0.00%	0.00%	0.03%	0.03%	0.02%
Flow (mL.min^-1^)							
- Chromatographic	0.8	1	1	1	1	1	0.1
- Post-column phase	0.25	6	3	1,2	4.5	2	0.5
% aqueous phase	1.5	87.9	75	54.5	82.4	66.7	83.3
Tubing L(m) × id(mm)	0.3 × 0.25	-	3 × 0.5	0.3 × 0.5	2 × 0.8	3 × 0.25	1.4 × 0.5
Time (s)	<1	60	9	2	11	3	27
Temperature (°C)	-	40	50	50	50	50	35

Post column DPH concentrations reported in the literature vary widely (Table 3). Optimizing of DPH concentration [54] showed that the DPH concentration has a weak influence on peak areas. With lower DPH concentrations, however, the range of linearity is smaller and background fluorescence is lower [54]. In practice, DPH should be used in micromolar concentrations, between 0.1 and 5 µmol·L^‑1^. 

### 4.5. Post-column flow rate

This parameter is crucial for determining the post-column dilution factor. Most post-column flow rates and most chromatographic/post-column flow-rate ratios, characteristics of mixing phase (polarity, viscosity), are decisive for detection. With DPH, the post column mixture contains between 55 and 88% water (v/v). A high water content is associated with lower background fluorescence but with high dilution. The post-column flow rate must be optimized and certainly depends on the composition of the organic phase.

### 4.6. Post column tubing length (or reaction time)

Post-column tubing is required for reaction, *i.e.,* formation *in situ* of small vesicles and DPH insertion. Tubing length usually varies from 0.3 to 3 m, but the inner diameter of tubing and flow-rate must also be considered. Finally, the reaction time is very short for BBOT (<1 s) and is from 2 to 60 seconds for DPH (Table 3). Fluorescence intensity decreases rapidly when tubing is either too short or too long. In addition, excessively long tubing should increase extra-column band broadening effects by diffusion [54].

### 4.7. Post-column system temperature

The post-column system temperature is classically set at a temperature similar to that of the chromatographic column, probably to limit perturbations during mixing of the chromatographic and post-column phases. The temperature of chromatographic separation modifies analysis time and resolution. A decrease of fluorescence intensity has been observed when the temperature increased from 25 °C to 45 °C [54]. When the temperature increases, molecular (Brownian) motion in fact favors the non-radiative deactivation of the first singlet state, resulting in a decreased quantum yield of the probe. In contrast, probe insertion in the supramolecular structure will be favored by an increase of temperature. The response of 16:0/16:0-GPCho was four-fold increased by increasing mixing temperature from 20 °C to 50 °C, but other phospholipids tested were not significantly affected by temperature [61]. This result suggests that insertion of molecular probe into the lamellar structure of liposomes depends on temperature for some phospholipids (with totally saturated long chain). These results are to be correlated to the gel or liquid state of the bilayer of the small unilamellar vesicule (SUV) formed, the temperature of the gel-fluid phase transition depending of the molecular structure of phospholipids. As a consequence, a compromise must be found that takes into account the temperature increase that favors insertion into the supramolecular association but that decreases the emission fluorescence of the probe.

### 4.8. Advantage of micro-LC with post-column device

One of the features of this type of detection is post-column dilution. As a result, post-column flow rates are very high, reaching 6 mL·min^-1^. To reduce flows rates, micro-LC could be used, without excessive miniaturization of the equipment. The advantages of micro-LC are well established [67] and make is possible to increase sensitivity and reduce solvent consumption [68]. The main problem involves the necessity of miniaturizing equipment and limiting band broadening due to contributions of the injection system, tubing connections and the detection cell [68,69]. The contribution from injection should be limited by staking injection [54]. The contribution from tubing (from dead volume) [68] could be limited by reducing the inner diameter of connections before mixing in the T-connector. After T-mixing, the resulting flow-rate must be compatible with conventional chromatographic equipment as a result of high post-column flow. In conclusion, this post-column device is compatible with micro-LC without the need for sophisticated equipment, in order to limit solvent consumption and to conductan efficient post-column detection.

## 5. Conclusions

This short review has concentrated on covalent derivatization and probe assisted detection methods used for the fluorescence detection of phospholipids in TLC, HPLC and CE. As shown, the first problem encountered with phospholipids is the absence of a chromophore in the compounds of interest. This results in limited possibilities for the analyst apart from the versatile use of mass spectrometry. Fluorescence detection with pre-column derivatization provides some advantages because of selectivity, large dynamic range and a suitable LOD resulting from chemical derivatization, whereas post-column supramolecular assemblies result in higher versatility as shown by the reported detection of other class of lipids, *i.e.,* triglycerides, ceramides or glycosphingolipids.
